# Cisplatin Decreases ENaC Activity Contributing to Renal Salt Wasting Syndrome

**DOI:** 10.3390/cancers12082140

**Published:** 2020-08-01

**Authors:** Antonio G. Soares, Elena Mironova, Crystal R. Archer, Jorge Contreras, James D. Stockand, Tarek Mohamed Abd El-Aziz

**Affiliations:** 1 Department of Cellular and Integrative Physiology, University of Texas Health Science Center at San Antonio, 7703 Floyd Curl Dr, San Antonio, TX 78229-3900, USA; soaresa@uthscsa.edu (A.G.S.); mironova@uthscsa.edu (E.M.); ArcherC@uthscsa.edu (C.R.A.); contrerasj12@uthscsa.edu (J.C.); mohamedt1@uthscsa.edu (T.M.A.E.-A.); 2 Zoology Department, Faculty of Science, Minia University, El-Minia 61519, Egypt

**Keywords:** Deg/ENaC channels, renal sodium excretion, hypertension, diuretic, pseduohypoaldosteronism, chemotherapy

## Abstract

Cisplatin (CDDP) is an important anticancer drug. A common side effect of CDDP is renal salt and water-wasting syndrome (RSWS). The origin of RSWS is obscure. Emerging evidence, though, suggests that broad inhibition of sodium transport proteins by CDDP may result in decreases in tubular reabsorption, causing increases in sodium and water excretion. In this sense, CDDP would be acting like a diuretic. The effect of CDDP on the epithelial Na^+^ channel (ENaC), which is the final arbiter fine-tuning renal Na^+^ excretion, is unknown. We test here whether CDDP affects ENaC to promote renal salt and water excretion. The effects of CDDP and benzamil (BZM), a blocker of ENaC, on excretion of a sodium load were quantified. Similar to BZM, CDDP facilitated renal Na^+^ excretion. To directly quantify the effects on ENaC, principal cells in split-open tubules were patch clamped. CDDP, at doses comparable to those used for chemotherapy (1.5 µM), significantly decreased ENaC activity in native tubules. To further elaborate on this mechanism, the dose-dependent effects of CDDP on mouse ENaC (mENaC) heterologously expressed in Chinese Hamster Ovary (CHO) cells were tested using patch clamping. As in native tubules, CDDP significantly decreased the activity of mENaC expressed in CHO cells. Dose–response curves and competition with amiloride identified CDDP as a weak inhibitor of ENaC (apparent IC_50_ = 1 µM) that competes with amiloride for inhibition of the channel, weakening the inhibitory actions of the latter. Such observations are consistent with CDDP being a partial modulator of ENaC, which possibly has a binding site that overlaps with that of amiloride. These findings are consistent with inhibition of ENaC by CDDP contributing to the RSWS caused by this important chemotherapy drug.

## 1. Introduction

Cisplatin (*cis*-diamminedichloroplatinum (II), or CDDP) and related compounds, including carboplatin and oxaliplatin, are platinum-containing drugs commonly used in chemotherapy. These drugs are powerful chemotherapy agents because of their ability to bind DNA and inhibit replication [1,2,3]. While important for chemotherapy, this class of drugs causes debilitating side effects, including renal salt and water-wasting syndrome (RSWS) and nephrotoxicity. It is unclear how these two complications of CDDP treatment are related. It is believed that the nephrotoxicity caused by CDDP results from DNA adduct formation, peroxidation of cell membranes, mitochondria dysfunction and apoptosis [2,4]. The origin of the RSWS caused by CDDP is more obscure.

Emerging evidence suggests that the RSWS caused by CDDP may result from accumulation in and dependent effects on the tubular epithelial cells, leading to an increase in urine output associated with natriuresis. CDDP is concentrated in proximal tubular epithelial cells via transport across the serosal membrane on the organic cation transporter 2 (OCT2) [5]. CDDP is secreted across the luminal membrane of proximal tubule epithelial cells and, consequently, concentrated in the urine in antiport to sodium via the multidrug and toxin extrusion protein 1 (SLC47A1) [6]. CDDP is elevated in the plasma and kidneys of mice lacking SLC47A1 [6]. In possible complement to the action in the proximal tubule, effects on epithelial cells in the loop of Henle or the distal nephron to include the collecting duct system have not been excluded.

In addition to damaging cells via direct actions on DNA, CDDP, independent of the effects on DNA, also binds to and is a general inhibitor of sodium transport proteins, including Na^+^/K^+^-ATPase [7,8]. Inhibition of such transport proteins in the kidney would cause diuresis associated with generalized salt wasting. Moreover, CDDP in rodents reduces the expression of aquaporin 2 (AQP2) and 3 [9,10]. Reduced expression of these two aquaporins, which set the water permeability of the distal nephron, could result in diuresis. However, this diuresis would be expected to be associated with dilute urine, which does not seem to be the case for CDDP-induced RSWS. Nonetheless, CDDP appears to decrease the activity of several proteins in the kidney involved in salt and water excretion. It is possible that the DNA-independent effects of CDDP on renal transport proteins could contribute to the RSWS caused by this drug.

The epithelial Na^+^ channel (ENaC) is a sodium-selective ion channel expressed in the luminal plasma membranes of late distal convoluted tubule, connecting tubule and collecting duct principal cells [11]. The coordinated action of ENaC and AQP2 in principal cells sets the sodium and water permeability of the collecting duct and, as such, act as the final arbiters, fine-tuning renal salt and water excretion. Inhibitors of ENaC, including amiloride and triamterene, are diuretics because they increase urine output as a function of increased sodium excretion [12]. It is unknown if CDDP affects ENaC. Blockers of ENaC though, including amiloride, function at high concentrations as general inhibitors of sodium transport proteins [13]. This is reminiscent of CDDP as possibly being a broad-spectrum inhibitor of sodium transport proteins. Moreover, it is worth noting that the guanidinium cation at the center of ENaC blockers, like benzamil (BZM) and amiloride, shares some similarity with the molecular structure and geometry of cisplatin [14]. Moreover, several heavy metals, including Cd^2+^, Cu^2+^, Hg^2+^, Ni^2+^, Pb^2+^ and Zn^2+^, are known to decrease the activity of ENaC [15]. It currently is unknown if ionized platinum affects ENaC.

Based on the understanding that CDDP causes RSWS, is concentrated in urine, has a general inhibitory effect on sodium transport proteins and has some chemical similarity with the guanidinium core of ENaC blockers, and that heavy metals affect the activity of ENaC, and that inhibitors of ENaC cause a natriuresis and diuresis similar to that caused by CDDP, we test here if CDDP affects ENaC activity and whether such action potentially could contribute to the RSWS caused by this widely used chemotherapy drug. Findings showed that CDDP causes a natriuresis similar to the ENaC blocker, BZM. In isolated tubules, CDDP rapidly decreases ENaC activity. In a heterologous expression system, CDDP decreases the activity of ENaC in a dose-dependent manner in a way that is competitive with the effects of amiloride. Together, these results are consistent with CDDP being a partial inhibitor of ENaC, with inhibition of this key renal ion channel possibly contributing to the RSWS caused by CDDP.

## 2. Results

### 2.1. CDDP Increases Urinary Sodium Excretion

**Figure 1:**
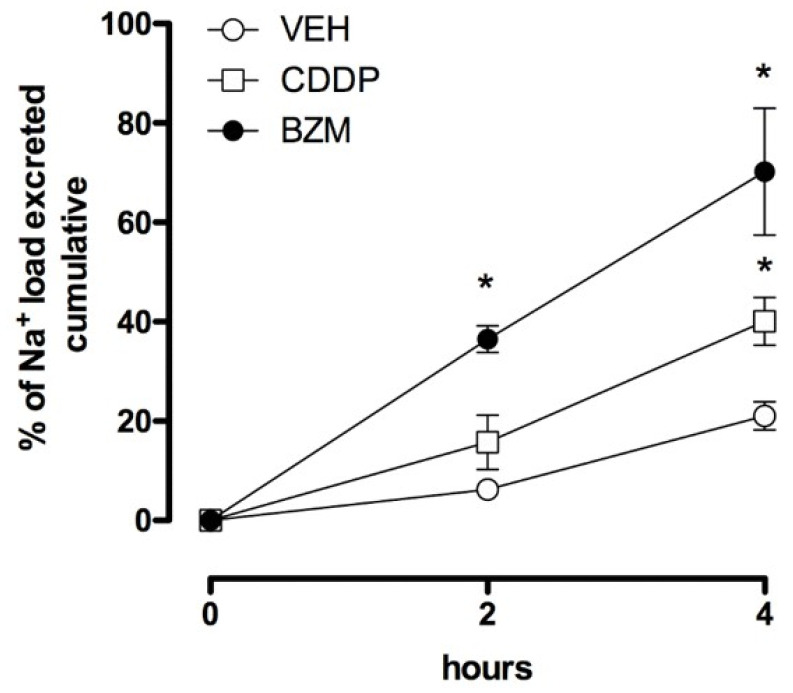
Cisplatin (CDDP) is natriuretic. Summary graph showing cumulative Na^+^ excretion in mice that received an Na^+^ load and treatment with cisplatin (CDDP, the black circles, where *n* = 5 independent trials with three mice per trial), benzamil (BZM, the white squares, where *n* = 5 independent trials with three mice per trial) or the vehicle (VEH, the white circles, where *n* = 5 independent trials with three mice per trial) over a 4 h period. Treatment with BZM significantly increased Na^+^ excretion at both the 2 h and 4 h time points. Treatment with CDDP significantly increased excretion at the 4 h time point, where BZM and CDDP were not different. * *p* < 0.05 vs. VEH group.

Male mice were housed socially at three mice/cage with free access to water and food. After 2 days, mice received an Na^+^ load (100 µL, 0.9% NaCl solution) plus treatment with CDDP, BZM or a vehicle. Excretion of this sodium load was then followed over the next four hours. Figure 1 shows the cumulative excretion of an Na^+^ load at 2 and 4 h following the injection of 100 µL of a 0.9% NaCl solution with BZM (1.4 mg/kg, intraperitoneal, i.p.), CDDP (25 mg/kg, i.p.) and a vehicle. At 2 h, the BZM group had significantly greater Na^+^ excretion compared with the saline-injected control group. At the 4 h time point, Na^+^ excretion was significantly greater in the CDDP and BZM groups compared with the control, with CDDP and BZM not being different. As expected, these results are consistent with CDDP, like BZM, facilitating Na^+^ excretion.

### 2.2. CDDP Reduces ENaC Activity in Split-Open Tubules

**Figure 2:**
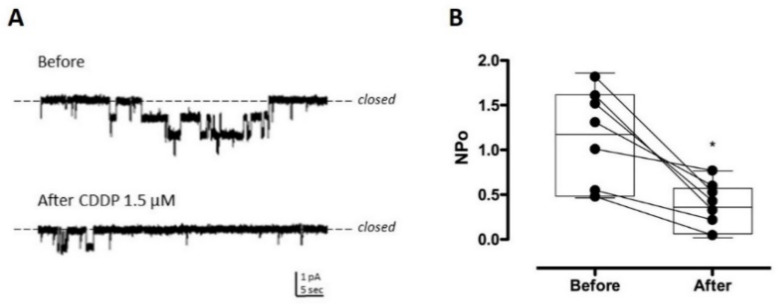
CDDP decreased the activity of the epithelial Na^+^ channel (ENaC) in native murine Principal cells. (**A**) Representative current traces of ENaC in cell-attached patches made on the apical plasma membrane of Principal cells in freshly isolated tubules from naïve mice before (top) and after (bottom) treatment with 1.5 µM CDDP. Negative pipette potential −60 mV and inward Na^+^ current downwards. (**B**) Summary graph of (*n* = 7) paired experiments showing ENaC activity (NP_o_) before and after treatment with CDDP. Summary data from experiments were identical to that shown in (A) * *p* < 0.05 vs. Before.

Split-open tubules isolated from the kidneys were prepared for single-channel patch clamp electrophysiology in the cell-attached configuration. Figure 2 shows representative continuous current traces from the patch clamped luminal plasma membranes of principal cells in isolated split-open tubules from the C57Bl/6 mouse. When ENaC activity was recorded in naïve tubules, the addition of CDDP rapidly reduced channel activity (NP_o_, 1.098 ± 0.19 vs. 0.3725 * ± 0.0917, before and after CDDP treatment, respectively, * *p* < 0.05 vs. Before). Figure 2A,B show representative traces ((A), with before above and after below) and summary effects (B), respectively, of the actions of CDDP (1.5 µM) on ENaC activity. These results are consistent with CDDP decreasing ENaC activity.

### 2.3. CDDP Is a Partial Inhibitor of ENaC

**Figure 3:**
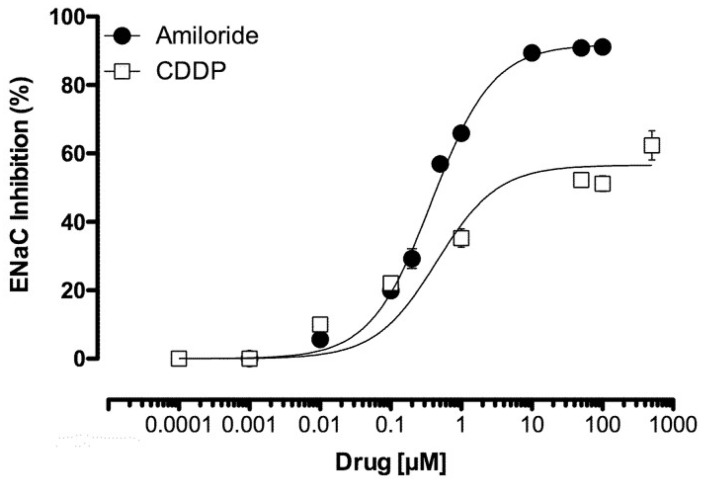
CDDP is a partial inhibitor of ENaC. Summary graph of the dose-dependent inhibitory effects of amiloride (black circles) and cisplatin (white boxes) on mENaC expressed in Chinese Hamster Ovary (CHO) cells (*n* = 3–5). CDDP significantly reduced ENaC activity with a reduced Emax compared with amiloride.

Chinese Hamster Ovary (CHO) cells expressing mENaC were patch clamped in the whole-cell configuration under voltage clamp conditions in the presence and absence of amiloride. Similar to the ENaC blocker amiloride, CDDP reduced mENaC activity in CHO cells in a dose-dependent manner. As reported in Figure 3, macroscopic mENaC currents were reduced with an IC_50_ of 0.46 ± 0.10 µM and 1.0 ± 0.18 µM for amiloride and CDDP, respectively. Maximal responses on mENaC activity, calculated as an inhibition percentage (Emax), were 91.9 ± 0.80 vs. 56.6 ± 4.2% for amiloride and CDDP, respectively, with the latter being significantly lower than the former. These results demonstrate that CDDP is a partial inhibitor of ENaC.

### 2.4. CDDP Competes with Amiloride for Inhibition of ENaC

**Figure 4:**
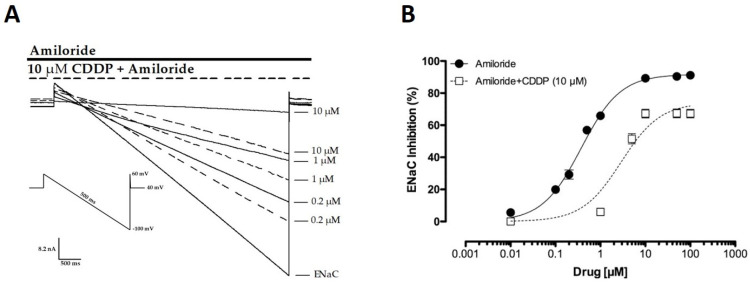
CDDP competes with amiloride, lessening the inhibitory effects of amiloride on ENaC. (**A**) Overlay of typical macroscopic current traces from a representative CHO cell expressing mENaC before and after treatment, with (0.2–10 µM) amiloride in the presence (dashed lines) and absence (full lines) of 10 µM of CDDP. Currents elicited by voltage ramps were stepped from a holding potential of 40 mV to 60 mV and ramped to −100 mV. (**B**) Summary graph of (*n* = 3–5 for each amiloride concentration) experiments showing concentration-dependent effects of amiloride on mENaC in the absence (black circles, full line) and presence (white boxes, dashed line) of 10 µM of CDDP. Exposure to CDDP reduced the maximal inhibition percentage of amiloride and caused a dextral displacement of the amiloride inhibition curve.

We noticed in patch clamp experiments that CDDP seemed to interfere with the amiloride block of the channel. To further explore this, we tested using patch clamp electrophysiology if CDDP competed with amiloride for a block of mENaC in an overexpression system using CHO cells. This was done by generating amiloride inhibition curves for mENaC in the presence of 10 µM of CDDP. Figure 4A shows representative macroscopic current traces of mENaC in the presence of amiloride and amiloride + CDDP. Figure 4B reports summary results of amiloride inhibition curves in the presence and absence of 10 µM of CDDP. Treatment with CDDP shifted the amiloride inhibition curve to the right, consistent with CDDP competing with amiloride for a block of the channel. Amiloride is an open channel blocker of the ENaC pore. CDDP showed a typical competitive modulator response with a dextral displacement of the effects of amiloride (IC_50_ = 499 ± 0.02 nM and 1.59 ± 0.19 µM for amiloride in the absence and presence of CDDP, where * *p* < 0.05 vs. amiloride) and a reduced Emax for amiloride (89.58 ± 1.26 vs. 67.48 ± 1.28 in the absence and presence of CDDP, respectively, where * *p* < 0.05).

## 3. Discussion

Our findings confirm that CDDP caused an expected natriuresis. In isolated tubules, CDDP rapidly decreased ENaC activity. In a heterologous expression system, CDDP decreased the activity of mENaC in a dose-dependent manner. The inhibition curve for CDDP on ENaC showed this drug to be a partial inhibitor of the channel. CDDP competed with amiloride for the inhibition of ENaC, reducing the effects of this more powerful inhibitor, which suggested that CDDP and amiloride may have overlapping binding sites on the channel.

Cisplatin is an important chemotherapy drug. Possible side effects of CDDP include RSWS [16,17,18,19]. As argued above (see Introduction), the inhibition of ENaC may potentially be contributing to the RSWS caused by CDDP. We began the current study by verifying that CDDP causes natriuresis. As supported by the results in Figure 1, CDDP caused a natriuresis similar to that elicited by the ENaC blocker, BZM. BZM, like all other inhibitors of ENaC, is a weak diuretic due to its ability to increase Na^+^ excretion as a consequence of inhibiting ENaC [20].

Exploring the mechanism underlying this CDDP-dependent natriuresis more deeply, we found, as supported by the results presented in Figure 2 and Figure 3, that CDDP inhibited ENaC in native distal nephron principal cells and in a heterologous expression system. Importantly, CDDP inhibited ENaC at doses consistent with those used for chemotherapy and causative for RSWS [16,21]. Such observations are consistent with this inhibitory action of CDDP on ENaC possibly being contributory to the RSWS caused by this chemotherapy drug.

CDDP was shown to be a partial inhibitor of ENaC in the current study, as supported by the results in Figure 3. As demonstrated by the results presented in Figure 4, CDDP competed with amiloride for the inhibition of ENaC. The latter is most consistent with CDDP and amiloride having, at least partially, overlapping binding sites on the channel. It is notable that amiloride is an open pore blocker of ENaC [20]. Future study will be needed to determine if CDDP also is a pore blocker of ENaC, though the current results strongly suggest that it may be.

A close inspection of the molecular structure of CDDP shows that this molecule shares some similarity with the pyrazinoyl guanidine group present in ENaC blockers, such as BZM and amiloride. Perhaps this explains how both could potentially be open channel blockers of ENaC that compete for a common or overlapping binding site. However, such contemplations are tempered by the need for further and more precise investigation of this possibility, including a precise mapping of both the amiloride and CDDP binding sites within ENaC.

An alternative explanation of a mechanism consistent with the observation that CDDP is a weak inhibitor of ENaC that competes with pore blockers of this ion channel is that CDDP contains platinum at its core, and ENaC is particularly susceptible to the effects of heavy metals. Many heavy metals, including Cd^2+^ and Hg^2+^, decrease ENaC activity [15]. However, others including Zn^2+^, Ni^2+^ and Cu^2+^ increase the activity of ENaC [11]. It is unclear as of yet if the effects of these heavy metals on ENaC are mediated by actions at the channel pore or if they are allosteric, mediated by a more distal binding site. If the former is true, then perhaps the platinum in CDDP is working in this regard. As stated above, such contemplations are tempered by the limitations of this study and the methods used here to assess CDDP’s effects on ENaC. Limitations include a lack of precise information about the specifics of possible direct CDDP interaction with ENaC. A feasible alternative to CDDP directly affecting ENaC is that CDDP influences ENaC activity secondarily to effects on other proteins, possibly including the Na^+^/K^+^-ATPase or the mineralocorticoid receptor. Inhibitory effects of CDDP on both of these proteins have been reported before [19,22].

While the current findings, due to limitations in methodology, cannot discount possible contribution from secondary effects of CDDP on ENaC, we believe the simplest interpretation of the current results is that, in addition to possible secondary effects mediated by inhibition of the Na^+^/K^+^-ATPase and the mineralocorticoid receptor, CDDP also likely has a more direct action on ENaC. Consequently, both the direct and secondary actions of CDDP on ENaC cause the RSWS associated with this important anticancer drug. We make this argument because CDDP affected ENaC in the current study in a heterologous system that did not include a mineralocorticoid receptor. In addition, the spatiotemporal effects of CDDP on ENaC in the current patch clamp study were rapid and localized to the plasma membrane. The effects of the mineralocorticoid receptor on ENaC are via transactivation localized to the nucleus, which manifests in minutes to hours. In the current study, CDDP rapidly reduced ENaC activity at the plasma membrane within seconds. Moreover, the competitive nature of CDDP and amiloride on ENaC are most consistent with, at least, some direct effect on the channel that is independent of secondary actions mediated by inhibition of the pump.

## 4. Materials and Methods

### 4.1. Animals

All animal use and welfare adhered to the National Institutes of Health Guide for the Care and Use of Laboratory Animals. Protocols were reviewed and approved by the Institutional Animal care and Use Committee of the University of Texas Health Science Center at San Antonio. Mice were housed and cared for in the Laboratory Animal Resources Facility at the University of Texas Health Science Center at San Antonio, which is fully accredited by the Association for Assessment and Accreditation of Laboratory Animal Care and licensed by the United States Department of Agriculture.

### 4.2. Metabolic Cage Experiments

Metabolic cage experiments followed previously published protocols with minor modifications [23]. Briefly, age and weight-matched C57Bl/6 male mice (~2 months old, 24.53 ± 0.15 g body weight) were randomly separated into 3 groups: (1) control, (2) CDDP and (3) BZM. Mice were housed socially at 3 mice/cage in metabolic cages (Techniplast, Buguggiate, Italy) and allowed to acclimate with free access to water and food for 2 days. On the third day, mice received an Na^+^ load (100 µL, 0.9% NaCl solution, i.p.) plus treatment with CDDP (25 mg/kg, i.p.), BZM (1.4 mg/kg, i.p.) or the vehicle. Urine volume and urinary Na^+^ concentration were evaluated every 2 h over a 4 h period. Urinary Na^+^ was quantified with a flame photometer (Jenway, Staffordshire, UK). Animals were observed during the whole time of the experimental period for clinical signs of pain or altered behavior. None were noticed.

### 4.3. Split-Open Tubule Preparation and Single-Channel Patch Clamp Electrophysiology

Split-open tubules amenable to patch clamp analysis were prepared as previously described [24]. Briefly, mouse kidneys were sectioned transversely. Segments of the connecting tubule (CNT) and cortical collecting duct (CCD) were manually microdissected with forceps and adhered to a glass chip coated with polylysine. These chips were transferred to an inverted microscope, where tubules were split open with sharpened pipettes. Single-channel patch clamp electrophysiology in the cell-attached configuration was then performed on the luminal plasma membranes of the principal cells in these split-opened tubules. The bath solution contained (in mM) 150 NaCl, 5 KCl, 1 CaCl_2_, 2 MgCl_2_, 5 glucose and 10 HEPES (pH 7.4) and the pipette solution contained (in mM) 140 LiCl, 2 MgCl_2_ and 10 HEPES (pH 7.4). Channel activity (open probability, P_o_, multiplied by channel number N) was calculated as previously described [24]. After recording baseline ENaC activity for 5 min, tubules were treated with CDDP (1.5 µM), with ENaC activity quantified in the presence of this drug over the next 5 min. This dose of CDDP showed no toxic effect during the experiments as cell capacitance and input resistances remained constant over the course of these experiments.

### 4.4. ENaC Expression in CHO Cells and Whole-Cell Patch Clamp Electrophysiology

CHO cells were maintained in a culture with Dulbecco’s modified Eagle’s medium, supplemented with 10% fetal bovine serum and 1% penicillin–streptomycin using standard methods [25]. Cells were plated on coverglass chips treated with 0.01% polylysine. Plated cells were transfected with plasmids encoding α-, β- and γ-mouse ENaC subunits genetically linked to NH_2_-terminal eCFP using a Polyfect reagent (Qiagen, Valencia, CA) as described previously [26,27]. In brief, 60% confluent cells in a 35 mm dish were treated with 2 μg of total plasmid cDNA for 24–48 h. Cells were maintained in a culture in the presence of 10 μM amiloride, replenished daily. Whole-cell macroscopic current recordings of mENaC expressed in CHO cells were made under voltage clamp conditions using standard methods [26,27,28]. In brief, the current through ENaC was the inward, amiloride-sensitive Na^+^ current with a bath solution of (in mM) 150 NaCl, 1 CaCl_2_, 2 MgCl_2_ and 10 HEPES (pH 7.4) and a pipette solution of (in mM) 120 CsCl, 5 NaCl, 2 MgCl_2_, 5 EGTA, 10 HEPES, 2 ATP and 0.1 GTP (pH 7.4). Current recordings were acquired with an Axopatch 200 B (Axon Instruments) patch clamp amplifier interfaced via a Digidata 1550 B (Axon Instruments) to a PC running the pClamp 11 suite of software (Axon Instruments). All currents were filtered at 1 kHz. Cells were clamped to a 40 mV holding potential with voltage ramps (500 ms) from 60 down to −100 mV, used to elicit current. ENaC activity is in the amiloride-sensitive current density at −80 mV. Whole-cell capacitance, on average 8–10 pF, was compensated. Series resistance, on average 3–6 megaohms, were also compensated. Cells expressing mENaC were evaluated by quantifying the macroscopic current in the presence and absence of amiloride. Dose–response curves for amiloride and CDDP were collected. In another set of experiments, cells were exposed to CDDP (10 µM) and a dose–response curve for amiloride was then collected. The EC_50_ and maximal response (Emax), expressed as inhibition (% inhibition) of channel activity, then were calculated. As stated above, this dose and the time course of CDDP showed no toxic effects for cell capacitance, and input resistances remained constant over the course of these experiments.

### 4.5. Statistics

Values were reported as mean ± standard error of the mean (SEM). Data were compared by a Student-t-test and a One-way ANOVA, followed by Bonferroni’s multiple comparisons test when appropriate and a *p* ≤ 0.05 was considered significant.

## 5. Conclusions

In conclusion, we demonstrated here that CDDP decreases ENaC activity, likely via a binding site on the channel that, at least partially, overlaps with that of amiloride. We also confirmed here that CDDP causes a natriuresis that is similar to that caused by ENaC blockers. ENaC blockers work as diuretics because they increase sodium excretion. We concluded that the direct inhibitory effect of CDDP on ENaC contributes to the RSWS caused by this important chemotherapy drug. This action of CDDP appears to be in addition to and separate from the effects of this drug on DNA replication, the mineralocorticoid receptor and Na^+^/K^+^-ATPase.
